# Effectiveness of sigmoidoscopy or colonoscopy screening on colorectal cancer incidence and mortality: a systematic review and meta-analysis of randomized controlled trial

**DOI:** 10.3389/fonc.2024.1364923

**Published:** 2024-03-14

**Authors:** Chunyang Han, Fan Wu, Jian Xu

**Affiliations:** Tongji Medical College, Huazhong University of Science and Technology, Wuhan, Hubei, China

**Keywords:** colorectal cancer, sigmoidoscopy, mortality, incidence, RCT

## Abstract

**Objectives:**

We conducted a comprehensive analysis to compare colonoscopy and sigmoidoscopy with standard care or fecal immunochemistry regarding colorectal cancer incidence and mortality risk.

**Methods:**

Until August 2023, literature from PubMed, Embase, Web of Science, and Cochrane was systematically reviewed. We examined the impact of colonoscopy or sigmoidoscopy versus standard care on colorectal cancer outcomes, including incidence, cancer-specific mortality, and overall mortality.

**Results:**

Among 4,265 screened articles, data from seven randomized controlled trials (involving 663,319 participants) were analyzed. The intervention group (colonoscopy or sigmoidoscopy) consisted of 258,938 participants, while the control group received standard care or fecal immunochemical testing, totaling 404,381 participants, with both groups having average colorectal cancer risk, without confounders. Pooled analyses indicated a 20% reduction in colorectal cancer incidence (RR: 0.80, 95% CI: 0.77-0.83) and a 26% decrease in colorectal cancer mortality (RR: 0.74, 95% CI: 0.69-0.80) in the intervention group compared to standard care. All-cause mortality remained unchanged (RR: 1.03, 95% CI: 0.99-1.07). Subgroup analysis favored sigmoidoscopy in reducing colorectal cancer morbidity and mortality.

**Conclusion:**

This meta-analysis of randomized controlled trials underscores the effectiveness of colonoscopy and, notably, sigmoidoscopy in reducing colorectal cancer incidence and mortality among average-risk populations. In comparison to fecal immunochemical testing, both colonoscopy and sigmoidoscopy did not significantly impact colorectal cancer incidence and mortality in this population.

**Systematic review registration:**

https://www.crd.york.ac.uk/PROSPERO/, identifier CRD42023460007.

## 1 Introduction

Colorectal cancer(CRC), a significant global health concern, stands as the third most commonly diagnosed cancer and the second leading cause of cancer-related deaths (1). For average-risk populations, mass screening with colonoscopy or sigmoidoscopy has been shown to have a large reduction in CRC incidence and mortality by detecting precancerous polyp production (2). However, the current long-term role of endoscopic screening is difficult to quantify due to the lack of pooled analyses of large multicenter randomized controlled trials with recent long-term follow-up.

Cancer screening currently serves two main purposes: early detection and prevention of cancer development (3). Treating cancer at an early stage, when it can be controlled and cured, reduces cancer mortality rates. Preventive cancer screening reduces long-term cancer-related illness and death by detecting and treating precancerous lesions like colorectal adenomas (4). Preventive screening serves as an efficient strategy for diminishing cancer occurrence and consequent fatalities (5).

In recent years, significant progress has been achieved in large randomized controlled trials (RCTs) aimed at evaluating various screening interventions for colorectal cancer. Notable examples include the UK Flexible Sigmoidoscopy Screening Trial (UKFSST) (6), the Norwegian Colorectal Cancer Prevention (NORCCAP) trial (7), the Prostate, Lung, Colorectal, and Ovarian Cancer (PLCO) trial (8), and the Nordic-European Initiative on Colorectal Cancer (NordICC) (9). These trials have contributed valuable data to our understanding of the effectiveness of different screening approaches in reducing the burden of colorectal cancer. Valuable data on the reduction of both the incidence and mortality rates of colorectal cancer have been gleaned from these trials, which were conducted over a median follow-up period of approximately 10 years or longer.

These large random controlled trials were conducted with healthy people at average risk of CRC (Excluding individuals with known potential risks for CRC) drawn by the experimenter from population registries in the area where the trial was conducted for a fixed time period, randomly assigned in a 1:1 or 1:2 ratio to either accept an invitation to undergo a single screening colonoscopy or a sigmoidoscopy (9, 10) (the invitation group) or not to accept an invitation (the no intervention group or to have a fecal immunochemical test (FIT) (11, 12). The main outcome measure assessed in the study was the incidence of colorectal cancer and mortality related to it, while the secondary outcome measure focused on death from any cause. Two previously published meta-analyses (13, 14) have examined the impact of sigmoidoscopy and FIT on CRC incidence and mortality. However, these analyses did not specifically investigate the effects of sigmoidoscopy or colonoscopy on CRC incidence and mortality, nor did they compare the effects of sigmoidoscopy or colonoscopy with FIT on CRC incidence and mortality. Since then, the outcomes of four randomized controlled trials have been published during the period 2020-2023 (6, 9–11). In this study, we provide a pooled analysis and updated evidence comparing the effectiveness of colonoscopy or sigmoidoscopy in reducing CRC incidence and mortality. Furthermore, we endeavor to assess the impact of colonoscopy or sigmoidoscopy in comparison with fecal immunochemistry on CRC outcomes.

## 2 Materials and methods

### 2.1 literature search

In this meta-analysis, we adhered to the guidelines outlined in the PRISMA 2020 statement (Preferred Reporting Items for Systematic Evaluation and Meta-Analysis 2020 statement) (15) and registered our study with PROSPERP under the registration number CRD42023460007. The PRISMA 2020 checklist can be found in **Supplementary Table 1** for your reference. As of August 2023, we used PubMed, Embase, Web of Science, and Cochrane to conduct a systematic literature search to compare the impact of colonoscopy or sigmoidoscopy on CRC morbidity or mortality, and published in English. Sigmoidoscopy”, “Colonoscopy”, “Colorectal Neoplasms”, “Incidence”, and “Mortality” as search terms to search the database. The detailed search strategy is provided in **Supplementary Table 1**. We also conducted a manual review of the bibliographies in all eligible studies. The retrieval and assessment of randomized controlled trials was conducted by two investigators independently. Differential results were assessed by a third investigator. If discrepancies are found during the literature search, the three researchers will negotiate to reach a consensus.

### 2.2 Identification of eligible studies

Studies were included if they met the following criteria.

(1) The study employed a randomized controlled trial design.
(2) The study population was at average risk of not having colorectal cancer.
(3) The intervention consisted of at least one of colonoscopy and sigmoidoscopy.
(4) The study findings included data on either colorectal cancer incidence rates or mortality rates, or both.
(5) Adequate data are available for the computation of the risk ratio (RR).

In our evaluation and analysis, we opted to exclude the following types of content: reviews, letters, comments, case reports, conference abstracts, unpublished articles, and non-English articles. These materials were excluded due to their limited availability of information for comprehensive assessment and analysis.

### 2.3 Data extraction

The search results were imported into the EndNote X9 database (Clarivate Analytics, Boston, MA, USA), and redundant entries were eliminated. Two researchers conducted the data extraction process independently. A third researcher dealt with differentiated results. Disagreements were resolved through consensus among the three researchers, and final decisions were reached according to standardized criteria. We have obtained the following information from the randomized controlled trials included in our analysis: primary author, publication year, duration of the study, geographic location of the study, study methodology, sample size, participant age, follow-up period, incidence of CRC mortality due to CRC, and overall mortality.

### 2.4 Quality assessment

The quality assessment of eligible RCTs was conducted following the Cochrane Handbook for Systematic Reviews of Interventions 5.1.0 based on seven terms: random sequence generation, allocation concealment, blinding of participants and personnel, blinding of outcome assessment, incomplete outcome data, selective reporting, and other sources of bias (16). Each facet of the study was categorized into three assessment outcomes: low risk, high risk, and unclear risk. Studies with a higher number of “low risk” bias assessments were considered to be of greater quality.

Both authors independently evaluated the quality of all the studies included in the analysis, and any discrepancies were resolved through constructive discussion.

### 2.5 Statistical analysis

Evidence synthesis was performed in Review Manager 5.4.1 version (Cochrane Collaboration, Oxford, UK). The relative risk (RR) was utilized to compare binary variables. All metrics were reported along with 95% confidence intervals (CIs). The variability among studies was evaluated using the chi-squared (χ²) test, commonly known as Cochran’s Q, and the inconsistency index (I²).

We considered statistical significance for heterogeneity as follows: A χ² p-value less than 0.05 or an I^2^ value exceeding 50% were regarded as indicative of substantial heterogeneity. In the presence of substantial heterogeneity (indicated by a χ² p-value < 0.05 or I² > 50%), a random-effects model was employed to calculate the pooled relative risk (RR). In an alternative approach, we utilized the fixed-effect model. we also conducted one-way sensitivity analyses to assess the impact of the included studies on the aggregated findings for outcomes displaying substantial heterogeneity. Publication bias was assessed through both visual inspection of funnel plots using Review Manager 5.3 (Cochrane Collaboration, Oxford, UK) and Egger’s regression tests conducted with Stata 12.0 (Stata Corp, College Station, TX, USA) for outcomes that included three or more studies (17). P value < 0.05 was considered as statistically significant publication bias.

## 3 Results

### 3.1 Literature search and study characteristics

**Figure 1:**
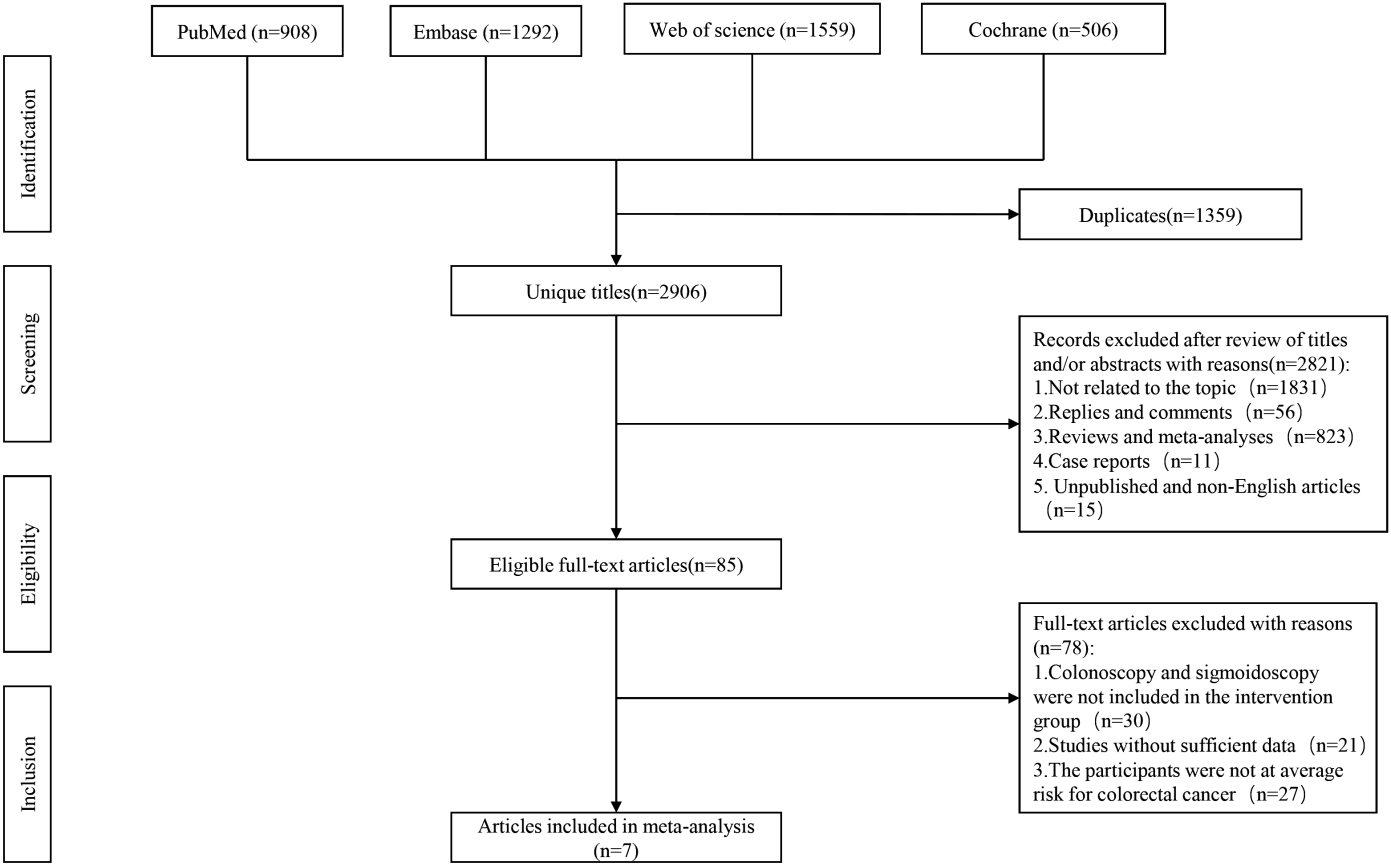
Flowchart of the systematic search and selection process.

**Figure 1** shows the flowchart of the systematic search, selection, and screening process. Through a comprehensive systematic literature search, a total of 4,265 pertinent articles were identified across various databases, including PubMed (n=908), Embase (n=1292), Web of Science (n=1559), and Cochrane (n=506). After eliminating redundant publications, a total of 2,906 titles and abstracts were subjected to review. Ultimately, our meta-analysis encompassed seven full-text articles that collectively comprised a participant pool of 663,319 individuals. It’s noteworthy that all seven of these studies were conducted as randomized controlled trials (**Table 1**).

**Table 1:** Basic features of the included literature.

Authors	Study period	Country	Study design	Registration number	Intervention	Control group	Patients (n)	Age (Years)	Median follow-up (Years)
							Scope/No intervention	Intervention/control	Incidence/Mortality
Quintero et al.	2008-2021	Spain	RCT	NCT00906997	Colonoscopy	Fit	5124/8976	59.1/59.3	10/10
Bretthauer et al.	2008-2021	Northern Europe	RCT	NCT00883792	Colonoscopy	No intervention	28220/56365		10/10
Randel et al.	2012-2019	Norway	RCT	NCT01538550	Sigmoidoscopy	Fit	69195/70096	63.3/52.2	10/10
Holme et al.	1999-2011	Norway	RCT	NCT00119912	Sigmoidoscopy	No intervention	20572/78220	56.1/56.9	10/10
Miller et al.	1993-2012	USA	RCT	NCT00002540	Sigmoidoscopy	No intervention	62312/62085		15.8
Atkin et al.	1995-2023	UK	RCT	ISRCTN28352761	Sigmoidoscopy	No intervention	56379/111503	60.0/60.0	17/17
Senore et al.	1995-2016	Italy	RCT	ISRCTN27814061	Sigmoidoscopy	No intervention	17136/17136		15.4/18.8

### 3.2 Quality assessment

The results of the quality assessment showed that the primary risk was from allocation concealment (selection bias), with almost all included RCTs showing high-risk outcomes, and the secondary risk was from blinding of subjects and experimental staff (implementation bias), with some studies showing high-risk outcomes. The included RCTs were all high-quality random controlled trials (**Supplementary Figures 1A, B**).

### 3.3 Subject characteristics

#### 3.3.1 Incidence

**Figure 2:**
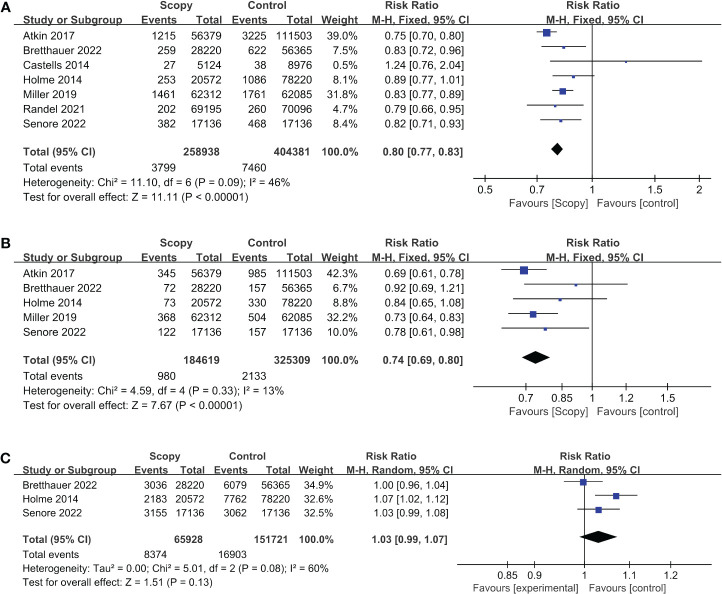
Forest plots of outcome indicator: **(A)** incidence of CRC, **(B) **mortality of CRC, **(C)** mortality for all causes.

In this study, we included a total of 7 RCTs, involving a cohort of 663,319 participants. Among them, 258,938 individuals were assigned to the intervention group, while the remaining 404,381 were allocated to the control group. The pooled analysis revealed a noteworthy decrease in the incidence of colorectal cancer within the intervention group [Relative Risk (RR) = 0.80, 95% Confidence Interval (CI) = 0.77-0.83]. Notably, no significant heterogeneity was detected among the included studies (I² = 46%, p < 0.00001) (**Figure 2A**). Upon visual inspection of funnel plots, a subtle indication of publication bias was observed; nevertheless, Egger’s test did not yield statistically significant results (p = 0.117) (**Supplementary Figure 2A**).

### 3.4 Mortality for colorectal cancer

In our analysis, we incorporated data from a comprehensive review comprising 5 RCTs, which collectively encompassed a sizable cohort of 509,928 study participants. Among these, 184,619 individuals were assigned to the intervention group, while 325,309 individuals constituted the control group. Our synthesis of the available evidence yielded a noteworthy finding-a statistically significant reduction in mortality rates associated with colorectal cancer among participants in the intervention group. The relative risk (RR) estimate was 0.74 (95% Confidence Interval [CI]: 0.69-0.80). Importantly, our analysis revealed minimal heterogeneity across the included studies (I²=13%, p<0.00001) (**Figure 2B**). The visual inspection of funnel plots revealed a minor indication of publication bias, while the results of Egger’s test indicated the existence of publication bias (p=0.014) (**Supplementary Figure 2B**).

### 3.5 Mortality for all causes

In this systematic review, a total of three RCTs, involving a cohort of 217,649 subjects were included. Among them, 65,928 were assigned to the intervention group, while 151,721 were in the control group. The results of our pooled analysis, illustrated in **Figure 2C** using a forest plot, revealed no statistically significant difference in all-cause mortality between the intervention and control groups, with a calculated risk ratio (RR) of 1.03 (95% CI, 0.99-1.07).

We noted a substantial level of heterogeneity among the included studies, as evidenced by an I² statistic of 60% and a p-value of 0.13. Furthermore, we conducted a comprehensive assessment of publication bias, both visually through funnel plots (**Supplementary Figure 2C**) and using Egger’s test, which yielded a non-significant result with a p-value of 0.350.

In summary, our analysis of these RCTs did not reveal a significant impact of the intervention on all-cause mortality. However, it’s important to acknowledge the presence of heterogeneity among the studies, and further investigation may be warranted to explore potential sources of variation.

### 3.6 Sensitivity analysis

We conducted a sensitivity analysis to evaluate the influence of individual RCTs on the analytical outcomes. This was achieved by systematically excluding each RCT, one at a time, from the analysis. The sensitivity analysis showed that the heterogeneity of the analytic results for CRC incidence and CRC mortality remained unchanged by excluding any individual RCT (**Supplementary Figures 3A, B**), whereas the heterogeneity for all-cause mortality disappeared when we excluded the data reported by Bretthauer et al. (9) in 2021 (I²=13%, p=0.009) (**Supplementary Figure 3C**), which suggests that the results of the present study for the analyses of CRC incidence and CRC mortality are stable. This suggests that the report explains the main reason for the heterogeneity in all-cause mortality rates.

### 3.7 Subgroup analysis

We performed a subgroup analysis of the intervention (colonoscopy or sigmoidoscopy) in the intervention group, and the treatment (FIT or no intervention) in the control group. The results of the subgroup analysis showed that sigmoidoscopy had a more significant effect on the reduction of CRC morbidity and mortality (INCIDENCE, RR, 0.79,95% CI, 0.76-0.83, I²=48%, p<0.03, MORTALITY FOR COLORECTAL CANCER RR, 0.73,95% CI. 0.67-0.79, I²=0%, p<0.00001) (**Table 2**). The effect of colonoscopy or sigmoidoscopy on long-term morbidity was stable and significant for CRC compared with the no intervention group (RR, 0.80,95% CI, 0.77-0.83), and because we did not include RCTs in which the control treatment was an immunochemical assay in the analysis of CRC mortality and all-cause mortality, we did not report the results of the subgroup analyses of the control treatments for CRC mortality and all-cause mortality. cause mortality subgroup analysis results.

**Table 2:** Results of subgroup analysis.

Subgroup	Incidence				Mortality for colorectal cancer				Mortality for all causes			
	Study	RR [95%CI]	P value	I^2^	Study	RR [95%CI]	P value	I^2^	Study	RR [95%CI]	P value	I^2^
Total	7	0.80 [0.77, 0.83]	<0.00001	46%	5	0.74 [0.69, 0.80]	<0.00001	13%	3	1.03 [0.99, 1.07]	0.13	60%
Intervention	7											
Colonoscopy	2	0.95 [0.65, 1.37]	0.77	58%	1	0.92 [0.69, 1.21]	0.54	NA	1	1.00 [0.96, 1.04]	0.91	NA
Sigmoidoscopy	5	0.79 [0.76, 0.83]	0.03	48%	4	0.73 [0.67, 0.79]	<0.00001	0%	2	1.05 [1.01, 1.09]	0.009	24%
Control group	7											
No intervention	5	0.80 [0.77, 0.83]	<0.00001	50%								
Fit	2	0.83 [0.70, 0.99]	0.75	66%								

RR, Risk ratio; CI, confidence interval; Fit, Fecal immunochemical test; NA, Not available.

## 4 Discussion

In pooled analyses, it was observed that screening methods involving sigmoidoscopy or colonoscopy led to a notable decrease of 20% in the incidence of CRC, with a relative risk (RR) of 0.80 [95% confidence interval (CI) 0.77-0.83], when compared to the usual-care group in the average-risk population. Additionally, there was a substantial 26% reduction in CRC-related mortality (RR, 0.74, 95% CI 0.69-0.80) associated with these screening approaches. However, it is important to note that there was no statistically significant change observed in all-cause mortality (RR, 1.03, 95% CI 0.99-1.07). The original authors of the relevant RCTs provided an explanation, suggesting that the slight increase in all-cause mortality might be associated with participants becoming more attentive to their health after engaging in the trial (9, 10, 18). The possibility of detecting diseases may have increased, leading to a mild elevation in all-cause mortality. Further subgroup analyses indicated that sigmoidoscopy had a more significant impact on reducing CRC morbidity and mortality.

Our pooled analysis of all seven randomized controlled trials assessed the long-term role of colorectal screening and the role of endoscopic screening compared with FIT. The results showed that colonoscopy or sigmoidoscopy, particularly sigmoidoscopy, was effective in reducing the incidence and mortality of colorectal cancer in an average-risk population. Nevertheless, in contrast to physical fitness, colonoscopy or sigmoidoscopy did not demonstrate a statistically significant reduction in the incidence and mortality rates of colorectal cancer among individuals at average risk. The quality assessment tables (**Supplementary Figure 1B**) and the associated pooled analyses suggest that the observed heterogeneity in all-cause mortality may be attributed to differences in trial design. Specifically, the randomized controlled trial conducted by Bretthauer et al. (2022) (9) employed a strict blinding protocol, wherein the purpose of the trial was concealed from patients, and the intervention group underwent tighter control, limiting their participation in other tests and behaviors beyond those specified by the experimenters. In contrast, in the random controlled trials reported by Holme et al. (18) and Senore et al. (10), the purpose of the trial was known to the intervention group and there was no monitoring of other relevant behaviors (19) (modification of lifestyle, other relevant tests).

Based on the data we extracted, the optimal age for endoscopic colorectal cancer screening is uncertain. Current guidelines recommend starting screening at age 45, 50, or 55 (20, 21). Based on expert consensus, it is recommended that sigmoidoscopy be performed every five years, while colonoscopy should be conducted every 10 years (22). Our study demonstrated long-term reductions in CRC morbidity and mortality after a single colonoscopy or sigmoidoscopy compared with the no intervention group, and although the intervention group participated in two sigmoidoscopies in the results reported by Miller et al. in 2019, sensitivity analyses showed that removing that RCT left our results almost unchanged (INCIDENCE, RR, 0.79,95% CI, 0.75-0.83, I²=49%, p<0.00001, mortality for colorectal cancer, RR, 0.75,95% CI, 0.68-0.82, I²=33%, p<0.00001),and Randel et al. reported in the 2021 results in a long-term reduction in the incidence of CRC after a single endoscopy compared with two rounds of Fit within two years (RR, 0.79,95% CI, 0.66-0.95), which may prompt guideline developers to reconsider the interval between endoscopic screenings (23), taking into account cost-effectiveness and available resources (5, 24).

The pooled analysis of the seven randomized controlled trials was based on an intention-to-treat analysis, indicating that selection bias was minimized (25). However, the true benefit of colonoscopy or sigmoidoscopy may be even greater than we reported due to adherence issues in the screening group and contamination interference in the no intervention group (26). Even using the latest methods of causal inference (27), it remains challenging to separate the actual screening benefits from analysis bias in each scenario. Therefore, we contend that the proposed intention-to-treat analyses provides the most effective results currently attainable.

Our subgroup analyses showed a more significant effect of sigmoidoscopy on morbidity and mortality of CRC (incidence, RR, 0.79,95% CI, 0.76-0.83, I²=48%, p<0.03, mortality for colorectal cancer RR, 0.73,95% CI. 0.67-0.79, I²=0%, p<0.00001). Compared with Fit, although the combined analysis showed that sigmoidoscopy or colonoscopy still had a more significant reduction in CRC morbidity and mortality, due to the heterogeneity of this outcome (I²=66%, p<0.75) and the fact that in the data reported by Castells et al. in 2014 (12) and Randel et al. in 2021 (11), respectively, data regarding a single endoscopy examination versus Fit examination varied considerably, and given the larger impact of the number of people actually examined in the intention-to-treat analyses and confounding factors such as the multiple rounds of examinations that may have been performed in the FIT group, the impact of endoscopy and Fit on CRC morbidity and mortality should be further combined with other factors (28) (e.g., exclusion of people included in the intervention group but who were not actually examined). The concept of endoscopy is similar to that of screening using fecal testing. Sigmoidoscopy or colonoscopy is itself a preventive screening tool through which polyps can be detected and removed, thereby reducing CRC incidence. This similarity leads us to analyze the impact of the two modalities of screening on CRC morbidity and mortality in terms of the resources available to society and the economic cost of screening (29, 30).

In the currently included RCTs, the geographical distribution is primarily concentrated in Europe and North America, with a notable lack of data from continents such as South America, Africa, and Asia. However, these continents are home to a large number of underdeveloped and developing countries and regions, where the protective role of colonoscopy or sigmoidoscopy for the local populations remains to be elucidated. More importantly, our data suggest that multiple rounds of FIT may approximate or achieve the protective effect against CRC provided by regular endoscopic examinations, such as colonoscopy every five years (22). In traditional randomized controlled trials (RCTs), single or double rounds of FIT are typically compared with endoscopic examination. However, directly equating a single round of FIT with endoscopic examination overlooks considerations of resource allocation efficiency and economic benefits (23).. In the future, designing RCT experiments that incorporate socio-economic factors (for example, scientifically adjusting the number of FIT rounds) will have greater clinical practice significance and societal value. Furthermore, we anticipate witnessing the emergence of RCTs with more rational and rigorous experimental designs and larger sample sizes. It is crucial to understand how to adjust the rounds of FIT to achieve or approximate the protective effects of colonoscopy or sigmoidoscopy within the same timeframe. Such studies could provide a cost-effective and well-protective alternative, particularly for resource-limited and economically challenged underdeveloped and developing countries and regions. This approach aims to intervene in the incidence and mortality rates of colorectal cancer globally, extending beyond the confines of Europe and North America.

The main strengths of our study are the large number of individuals included, the long-term follow-up, the access to previously unpublished detailed data, and the expertise of all study groups. Endoscopy (including both colonoscopy and sigmoidoscopy) was analyzed as a comprehensive entity, and compared with FIT. Limitations of this analysis include the relatively small number of included randomized controlled trials (7 RCTs). Additionally, while the Akita trial in Japan (31) is of interest, it has only reported baseline results thus far. Given this limitation, our analysis primarily focused on RCTs conducted in Europe and North America. However, it is important to note that data from other continents, such as Asia and Africa, are lacking in this analysis.

In conclusion, our pooled analysis of all seven large randomized trials on sigmoidoscopy or colonoscopy screening suggests that both procedures have a significant impact on colorectal cancer (CRC) morbidity and mortality. They can effectively reduce long-term CRC morbidity and mortality. Based on our analysis of the data and baseline information from the RCTs included in our study, and taking into account resource allocation efficiency, we recommend initiating sigmoidoscopy screening at age 50 and repeating it every three to five years, alongside colonoscopy screening every five years.

Regarding colonoscopy compared to FIT, further discussion is needed on their impact on colorectal cancer incidence and mortality, taking into account a variety of factors such as the number of testing rounds and the invasiveness of the testing modality. However, based on our data analysis results and the current limited experimental designs of RCTs, while prioritizing resource and economic considerations, we recommend annual FIT for individuals aged 50 and above.

## Data availability statement

The original contributions presented in the study are included in the article/**Supplementary Material**. Further inquiries can be directed to the corresponding author.

## Author contributions

CH: Conceptualization, Data curation, Methodology, Resources, Writing – original draft, Writing – review & editing. FW: Conceptualization, Data curation, Methodology, Supervision, Writing – original draft, Writing – review & editing. JX: Formal analysis, Resources, Software, Supervision, Validation, Writing – original draft.

## References

[1] Sung H Ferlay J Siegel RL Laversanne M Soerjomataram I Jemal A . Global cancer statistics 2020: GLOBOCAN estimates of incidence and mortality worldwide for 36 cancers in 185 countries. CA Cancer J Clin. (2021) 71:209–49. doi: 10.3322/caac.21660 33538338

[2] Chan SCH Liang JQ . Advances in tests for colorectal cancer screening and diagnosis. Expert Rev Mol Diagn. (2022) 22:449–60. doi: 10.1080/14737159.2022.2065197 35400293

[3] Wolf AMD Fontham ETH Church TR Flowers CR Guerra CE LaMonte SJ . Colorectal cancer screening for average-risk adults: 2018 guideline update from the American Cancer Society. CA Cancer J Clin. (2018) 68:250–81. doi: 10.3322/caac.21457 29846947

[4] Ladabaum U Dominitz JA Kahi C Schoen RE . Strategies for colorectal cancer screening. Gastroenterology. (2020) 158:418–32. doi: 10.1053/j.gastro.2019.06.043 31394083

[5] Shaukat A Levin TR . Current and future colorectal cancer screening strategies. Nat Rev Gastroenterol Hepatol. (2022) 19:521–31. doi: 10.1038/s41575-022-00612-y PMC9063618 35505243

[6] Cross AJ Robbins EC Saunders BP Duffy SW Wooldrage K . Higher adenoma detection rates at screening associated with lower long-term colorectal cancer incidence and mortality. Clin Gastroenterol Hepatol. (2022) 20:e148–67. doi: 10.1016/j.cgh.2020.09.020 PMC8811539 32931959

[7] Holme Ø Løberg M Kalager M Bretthauer M Hernán MA Aas E . Long-term effectiveness of sigmoidoscopy screening on colorectal cancer incidence and mortality in women and men: A randomized trial. Ann Intern Med. (2018) 168:775–82. doi: 10.7326/M17-1441 PMC6853067 29710125

[8] Miller EA Pinsky PF Schoen RE Prorok PC Church TR . Effect of flexible sigmoidoscopy screening on colorectal cancer incidence and mortality: long-term follow-up of the randomised US PLCO cancer screening trial. Lancet Gastroenterol Hepatol. (2019) 4:101–10. doi: 10.1016/S2468-1253(18)30358-3 PMC6335177 30502933

[9] Bretthauer M Løberg M Wieszczy P Kalager M Emilsson L Garborg K . Effect of colonoscopy screening on risks of colorectal cancer and related death. N Engl J Med. (2022) 387:1547–56. doi: 10.1056/NEJMoa2208375 36214590

[10] Senore C Riggi E Armaroli P Bonelli L Sciallero S Zappa M . Long-term follow-up of the italian flexible sigmoidoscopy screening trial. Ann Intern Med. (2022) 175:36–45. doi: 10.7326/M21-0977 34748376

[11] Randel KR Schult AL Botteri E Hoff G Bretthauer M Ursin G . Colorectal cancer screening with repeated fecal immunochemical test versus sigmoidoscopy: baseline results from a randomized trial. Gastroenterology. (2021) 160:1085–1096.e5. doi: 10.1053/j.gastro.2020.11.037 33227280

[12] Castells A Quintero E . Programmatic screening for colorectal cancer: the COLONPREV study. Dig Dis Sci. (2015) 60:672–80. doi: 10.1007/s10620-014-3446-2 25492501

[13] Juul FE Cross AJ Schoen RE Senore C Pinsky P Miller E . 15-year benefits of sigmoidoscopy screening on colorectal cancer incidence and mortality : A pooled analysis of randomized trials. Ann Intern Med. (2022) 175:1525–33. doi: 10.7326/M22-0835 36215714

[14] Zhang C Liu L Li J Lv Y Wu D Xu S . Effect of flexible sigmoidoscopy-based screening on colorectal cancer incidence and mortality: an updated systematic review and meta-analysis of randomized controlled trials. Expert Rev Anticancer Ther. (2023) 23:1217–27. doi: 10.1080/14737140.2023.2245564 37542427

[15] Page MJ McKenzie JE Bossuyt PM Boutron I Hoffmann TC Mulrow CD . The PRISMA 2020 statement: an updated guideline for reporting systematic reviews. BMJ. (2021) 372:n71. doi: 10.1136/bmj.n71 33782057 PMC8005924

[16] Cumpston M Li T Page MJ Chandler J Welch VA Higgins JP . Updated guidance for trusted systematic reviews: a new edition of the Cochrane Handbook for Systematic Reviews of Interventions. Cochrane Database Syst Rev. (2019) 10:ED000142. doi: 10.1002/14651858.ED000142 31643080 PMC10284251

[17] Egger M Davey Smith G Schneider M Minder C . Bias in meta-analysis detected by a simple, graphical test. BMJ. (1997) 315:629–34. doi: 10.1136/bmj.315.7109.629 PMC2127453 9310563

[18] Holme Ø Løberg M Kalager M Bretthauer M Hernán MA Aas E . Effect of flexible sigmoidoscopy screening on colorectal cancer incidence and mortality: a randomized clinical trial. JAMA. (2014) 312:606–15. doi: 10.1001/jama.2014.8266 PMC4495882 25117129

[19] Gondal G Grotmol T Hofstad B Bretthauer M Eide TJ Hoff G . Lifestyle-related risk factors and chemoprevention for colorectal neoplasia: experience from the large-scale NORCCAP screening trial. Eur J Cancer Prev. (2005) 14:373–9. doi: 10.1097/00008469-200508000-00010 16030428

[20] Helsingen LM Vandvik PO Jodal HC Agoritsas T Lytvyn L Anderson JC . Colorectal cancer screening with faecal immunochemical testing, sigmoidoscopy or colonoscopy: a clinical practice guideline. BMJ. (2019) 367:l5515. doi: 10.1136/bmj.l5515 31578196

[21] Patel SG May FP Anderson JC Burke CA Dominitz JA Gross SA . Updates on age to start and stop colorectal cancer screening: recommendations from the U.S. Multi-society task force on colorectal cancer. Am J Gastroenterol. (2022) 117:57–69. doi: 10.14309/ajg.0000000000001548 34962727

[22] Davidson KW Barry MJ Mangione CM Cabana M Caughey AB Davis EM . Screening for colorectal cancer: US preventive services task force recommendation statement. JAMA. (2021) 325:1965–77. doi: 10.1001/jama.2021.6238 34003218

[23] Lin JS Perdue LA Henrikson NB Bean SI Blasi PR . Screening for colorectal cancer: updated evidence report and systematic review for the US preventive services task force. JAMA. (2021) 325:1978–98. doi: 10.1001/jama.2021.4417 PMC13509038 34003220

[24] Sharp L Tilson L Whyte S O'Ceilleachair A Walsh C Usher C . Cost-effectiveness of population-based screening for colorectal cancer: a comparison of guaiac-based faecal occult blood testing, faecal immunochemical testing and flexible sigmoidoscopy. Br J Cancer. (2012) 106:805–16. doi: 10.1038/bjc.2011.580 PMC3305953 22343624

[25] Akl EA Sun X Busse JW Johnston BC Briel M Mulla S . Specific instructions for estimating unclearly reported blinding status in randomized trials were reliable and valid. J Clin Epidemiol. (2012) 65:262–7. doi: 10.1016/j.jclinepi.2011.04.015 22200346

[26] Higgins JP Thompson SG Deeks JJ Altman DG . Measuring inconsistency in meta-analyses. Bmj. (2003) 327:557–60. doi: 10.1136/bmj.327.7414.557 PMC192859 12958120

[27] Acar O Nazlican E Dönmez E . Colorectal cancer screening: understanding the needs of the pre-screening group. Cent Eur J Public Health. (2023) 31:3–8. doi: 10.21101/cejph.a7184 36976250

[28] Pox CP . Controversies in colorectal cancer screening. Digestion. (2014) 89:274–81. doi: 10.1159/000363287 25034478

[29] Areia M Fuccio L Hassan C Dekker E Dias-Pereira A Dinis-Ribeiro M . Cost-utility analysis of colonoscopy or faecal immunochemical test for population-based organised colorectal cancer screening. United Eur Gastroenterol J. (2019) 7:105–13. doi: 10.1177/2050640618803196 PMC6374854 30788122

[30] Tinmouth J Lansdorp-Vogelaar I Allison JE . Faecal immunochemical tests versus guaiac faecal occult blood tests: what clinicians and colorectal cancer screening programme organisers need to know. Gut. (2015) 64:1327–37. doi: 10.1136/gutjnl-2014-308074 26041750

[31] Saito H Kudo SE Takahashi N Yamamoto S Kodama K Nagata K . Efficacy of screening using annual fecal immunochemical test alone versus combined with one-time colonoscopy in reducing colorectal cancer mortality: the Akita Japan population-based colonoscopy screening trial (Akita pop-colon trial). Int J Colorectal Dis. (2020) 35:933–9. doi: 10.1007/s00384-020-03518-w 32034490

