# Supplementary material for: Effectiveness of sigmoidoscopy or colonoscopy screening on colorectal cancer incidence and mortality: a systematic review and meta-analysis of randomized controlled trial
Source: Front Oncol. 2024 Mar 14;14:1364923. doi: 10.3389/fonc.2024.1364923 (PMC10972922; doi:10.3389/fonc.2024.1364923)
Supplement: Supplementary file 1 [file DataSheet_1.docx]

**Supplementary materials**

**Table S1.** The searching strategy

1. Pubmed-908

((((("Sigmoidoscopy"[Mesh]) OR (((((((((((((Sigmoidoscopies) OR (Proctosigmoidoscopy)) OR (Proctosigmoidoscopies)) OR (Sigmoidoscopic Surgical Procedures)) OR (Procedure, Sigmoidoscopic Surgical)) OR (Procedures, Sigmoidoscopic Surgical)) OR (Sigmoidoscopic Surgical Procedure)) OR (Surgical Procedure, Sigmoidoscopic)) OR (Surgery, Sigmoidoscopic)) OR (Surgical Procedures, Sigmoidoscopic)) OR (Sigmoidoscopic Surgery)) OR (Sigmoidoscopic Surgeries)) OR (Surgeries, Sigmoidoscopic))) OR (("Colonoscopy"[Mesh]) OR (((((((((((Colonoscopies) OR (Colonoscopic Surgical Procedures)) OR (Colonoscopic Surgical Procedure)) OR (Procedure, Colonoscopic Surgical)) OR (Procedures, Colonoscopic Surgical)) OR (Surgical Procedure, Colonoscopic)) OR (Surgery, Colonoscopic)) OR (Surgical Procedures, Colonoscopic)) OR (Colonoscopic Surgery)) OR (Colonoscopic Surgeries)) OR (Surgeries, Colonoscopic)))) AND (("Colorectal Neoplasms"[Mesh]) OR (((((((((((((((Colorectal Neoplasm) OR (Neoplasm, Colorectal)) OR (Neoplasms, Colorectal)) OR (Colorectal Tumors)) OR (Colorectal Tumor)) OR (Tumor, Colorectal)) OR (Tumors, Colorectal)) OR (Colorectal Cancer)) OR (Cancer, Colorectal)) OR (Cancers, Colorectal)) OR (Colorectal Cancers)) OR (Colorectal Carcinoma)) OR (Carcinoma, Colorectal)) OR (Carcinomas, Colorectal)) OR (Colorectal Carcinomas)))) AND (((RCT) OR (randomized controlled trial)) OR (clinical trail))) AND ((("Incidence"[Mesh]) OR (((((((((((((((((((((Incidences) OR (Secondary Attack Rate)) OR (Attack Rate, Secondary)) OR (Rate, Secondary Attack)) OR (Secondary Attack Rates)) OR (Incidence Proportion)) OR (Incidence Proportions)) OR (Proportion, Incidence)) OR (Attack Rate)) OR (Attack Rates)) OR (Rate, Attack)) OR (Cumulative Incidence)) OR (Cumulative Incidences)) OR (Incidence, Cumulative)) OR (Incidence Rate)) OR (Incidence Rates)) OR (Rate, Incidence)) OR (Person-time Rate)) OR (Person time Rate)) OR (Person-time Rates)) OR (Rate, Person-time))) OR (("Mortality"[Mesh]) OR ((((((((((((((((((((((((((((((((((((((Mortalities) OR (Case Fatality Rate)) OR (Case Fatality Rates)) OR (Rate, Case Fatality)) OR (Rates, Case Fatality)) OR (CFR Case Fatality Rate)) OR (Crude Death Rate)) OR (Crude Death Rates)) OR (Death Rate, Crude)) OR (Rate, Crude Death)) OR (Crude Mortality Rate)) OR (Crude Mortality Rates)) OR (Mortality Rate, Crude)) OR (Rate, Crude Mortality)) OR (Death Rate)) OR (Death Rates)) OR (Rate, Death)) OR (Mortality Rate)) OR (Mortality Rates)) OR (Rate, Mortality)) OR (Mortality, Excess)) OR (Excess Mortality)) OR (Excess Mortalities)) OR (Decline, Mortality)) OR (Mortality Declines)) OR (Mortality Decline)) OR (Mortality Determinants)) OR (Determinants, Mortality)) OR (Determinant, Mortality)) OR (Mortality Determinant)) OR (Mortality, Differential)) OR (Differential Mortality)) OR (Differential Mortalities)) OR (Age-Specific Death Rate)) OR (Age-Specific Death Rates)) OR (Death Rate, Age-Specific)) OR (Rate, Age-Specific Death)) OR (Age Specific Death Rate))))

2. Embase-1292/Cochrane-506

(((((Sigmoidoscopy) OR (((((((((((((Sigmoidoscopies) OR (Proctosigmoidoscopy)) OR (Proctosigmoidoscopies)) OR (Sigmoidoscopic Surgical Procedures)) OR (Procedure, Sigmoidoscopic Surgical)) OR (Procedures, Sigmoidoscopic Surgical)) OR (Sigmoidoscopic Surgical Procedure)) OR (Surgical Procedure, Sigmoidoscopic)) OR (Surgery, Sigmoidoscopic)) OR (Surgical Procedures, Sigmoidoscopic)) OR (Sigmoidoscopic Surgery)) OR (Sigmoidoscopic Surgeries)) OR (Surgeries, Sigmoidoscopic))) OR ((Colonoscopy) OR (((((((((((Colonoscopies) OR (Colonoscopic Surgical Procedures)) OR (Colonoscopic Surgical Procedure)) OR (Procedure, Colonoscopic Surgical)) OR (Procedures, Colonoscopic Surgical)) OR (Surgical Procedure, Colonoscopic)) OR (Surgery, Colonoscopic)) OR (Surgical Procedures, Colonoscopic)) OR (Colonoscopic Surgery)) OR (Colonoscopic Surgeries)) OR (Surgeries, Colonoscopic)))) AND ((Colorectal Neoplasms) OR (((((((((((((((Colorectal Neoplasm) OR (Neoplasm, Colorectal)) OR (Neoplasms, Colorectal)) OR (Colorectal Tumors)) OR (Colorectal Tumor)) OR (Tumor, Colorectal)) OR (Tumors, Colorectal)) OR (Colorectal Cancer)) OR (Cancer, Colorectal)) OR (Cancers, Colorectal)) OR (Colorectal Cancers)) OR (Colorectal Carcinoma)) OR (Carcinoma, Colorectal)) OR (Carcinomas, Colorectal)) OR (Colorectal Carcinomas)))) AND (((RCT) OR (randomized controlled trial)) OR (clinical trial))) AND (((Incidence) OR (((((((((((((((((((((Incidences) OR (Secondary Attack Rate)) OR (Attack Rate, Secondary)) OR (Rate, Secondary Attack)) OR (Secondary Attack Rates)) OR (Incidence Proportion)) OR (Incidence Proportions)) OR (Proportion, Incidence)) OR (Attack Rate)) OR (Attack Rates)) OR (Rate, Attack)) OR (Cumulative Incidence)) OR (Cumulative Incidences)) OR (Incidence, Cumulative)) OR (Incidence Rate)) OR (Incidence Rates)) OR (Rate, Incidence)) OR (Person-time Rate)) OR (Person time Rate)) OR (Person-time Rates)) OR (Rate, Person-time))) OR ((Mortality) OR ((((((((((((((((((((((((((((((((((((((Mortalities) OR (Case Fatality Rate)) OR (Case Fatality Rates)) OR (Rate, Case Fatality)) OR (Rates, Case Fatality)) OR (CFR Case Fatality Rate)) OR (Crude Death Rate)) OR (Crude Death Rates)) OR (Death Rate, Crude)) OR (Rate, Crude Death)) OR (Crude Mortality Rate)) OR (Crude Mortality Rates)) OR (Mortality Rate, Crude)) OR (Rate, Crude Mortality)) OR (Death Rate)) OR (Death Rates)) OR (Rate, Death)) OR (Mortality Rate)) OR (Mortality Rates)) OR (Rate, Mortality)) OR (Mortality, Excess)) OR (Excess Mortality)) OR (Excess Mortalities)) OR (Decline, Mortality)) OR (Mortality Declines)) OR (Mortality Decline)) OR (Mortality Determinants)) OR (Determinants, Mortality)) OR (Determinant, Mortality)) OR (Mortality Determinant)) OR (Mortality, Differential)) OR (Differential Mortality)) OR (Differential Mortalities)) OR (Age-Specific Death Rate)) OR (Age-Specific Death Rates)) OR (Death Rate, Age-Specific)) OR (Rate, Age-Specific Death)) OR (Age Specific Death Rate))))

3. WOS-1559

(((((Sigmoidoscopy) OR (((((((((((((Sigmoidoscopies) OR (Proctosigmoidoscopy)) OR (Proctosigmoidoscopies)) OR (Sigmoidoscopic Surgical Procedures)) OR (Procedure, Sigmoidoscopic Surgical)) OR (Procedures, Sigmoidoscopic Surgical)) OR (Sigmoidoscopic Surgical Procedure)) OR (Surgical Procedure, Sigmoidoscopic)) OR (Surgery, Sigmoidoscopic)) OR (Surgical Procedures, Sigmoidoscopic)) OR (Sigmoidoscopic Surgery)) OR (Sigmoidoscopic Surgeries)) OR (Surgeries, Sigmoidoscopic))) OR ((Colonoscopy) OR (((((((((((Colonoscopies) OR (Colonoscopic Surgical Procedures)) OR (Colonoscopic Surgical Procedure)) OR (Procedure, Colonoscopic Surgical)) OR (Procedures, Colonoscopic Surgical)) OR (Surgical Procedure, Colonoscopic)) OR (Surgery, Colonoscopic)) OR (Surgical Procedures, Colonoscopic)) OR (Colonoscopic Surgery)) OR (Colonoscopic Surgeries)) OR (Surgeries, Colonoscopic)))) AND ((Colorectal Neoplasms) OR (((((((((((((((Colorectal Neoplasm) OR (Neoplasm, Colorectal)) OR (Neoplasms, Colorectal)) OR (Colorectal Tumors)) OR (Colorectal Tumor)) OR (Tumor, Colorectal)) OR (Tumors, Colorectal)) OR (Colorectal Cancer)) OR (Cancer, Colorectal)) OR (Cancers, Colorectal)) OR (Colorectal Cancers)) OR (Colorectal Carcinoma)) OR (Carcinoma, Colorectal)) OR (Carcinomas, Colorectal)) OR (Colorectal Carcinomas)))) AND (((RCT) OR (randomized controlled trial)) OR (clinical trial))) AND (((Incidence) OR (((((((((((((((((((((Incidences) OR (Secondary Attack Rate)) OR (Attack Rate, Secondary)) OR (Rate, Secondary Attack)) OR (Secondary Attack Rates)) OR (Incidence Proportion)) OR (Incidence Proportions)) OR (Proportion, Incidence)) OR (Attack Rate)) OR (Attack Rates)) OR (Rate, Attack)) OR (Cumulative Incidence)) OR (Cumulative Incidences)) OR (Incidence, Cumulative)) OR (Incidence Rate)) OR (Incidence Rates)) OR (Rate, Incidence)) OR (Person-time Rate)) OR (Person time Rate)) OR (Person-time Rates)) OR (Rate, Person-time))) OR ((Mortality) OR ((((((((((((((((((((((((((((((((((((((Mortalities) OR (Case Fatality Rate)) OR (Case Fatality Rates)) OR (Rate, Case Fatality)) OR (Rates, Case Fatality)) OR (CFR Case Fatality Rate)) OR (Crude Death Rate)) OR (Crude Death Rates)) OR (Death Rate, Crude)) OR (Rate, Crude Death)) OR (Crude Mortality Rate)) OR (Crude Mortality Rates)) OR (Mortality Rate, Crude)) OR (Rate, Crude Mortality)) OR (Death Rate)) OR (Death Rates)) OR (Rate, Death)) OR (Mortality Rate)) OR (Mortality Rates)) OR (Rate, Mortality)) OR (Mortality, Excess)) OR (Excess Mortality)) OR (Excess Mortalities)) OR (Decline, Mortality)) OR (Mortality Declines)) OR (Mortality Decline)) OR (Mortality Determinants)) OR (Determinants, Mortality)) OR (Determinant, Mortality)) OR (Mortality Determinant)) OR (Mortality, Differential)) OR (Differential Mortality)) OR (Differential Mortalities)) OR (Age-Specific Death Rate)) OR (Age-Specific Death Rates)) OR (Death Rate, Age-Specific)) OR (Rate, Age-Specific Death)) OR (Age Specific Death Rate)))) (Topic) and Preprint Citation Index (Exclude – Database)


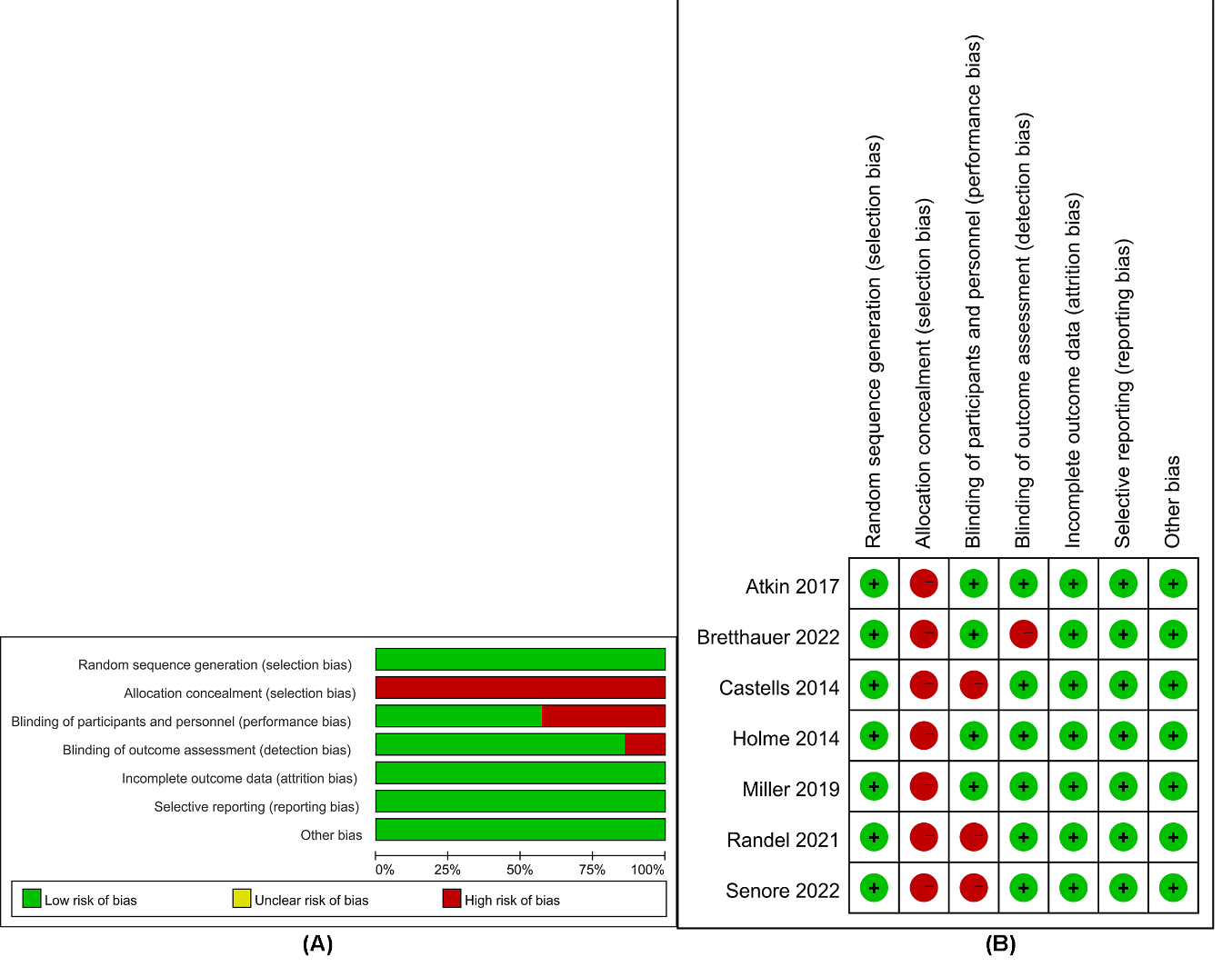


**Fig S1. (A)** Risk of bias gragh; **(B)**Risks of bias summary


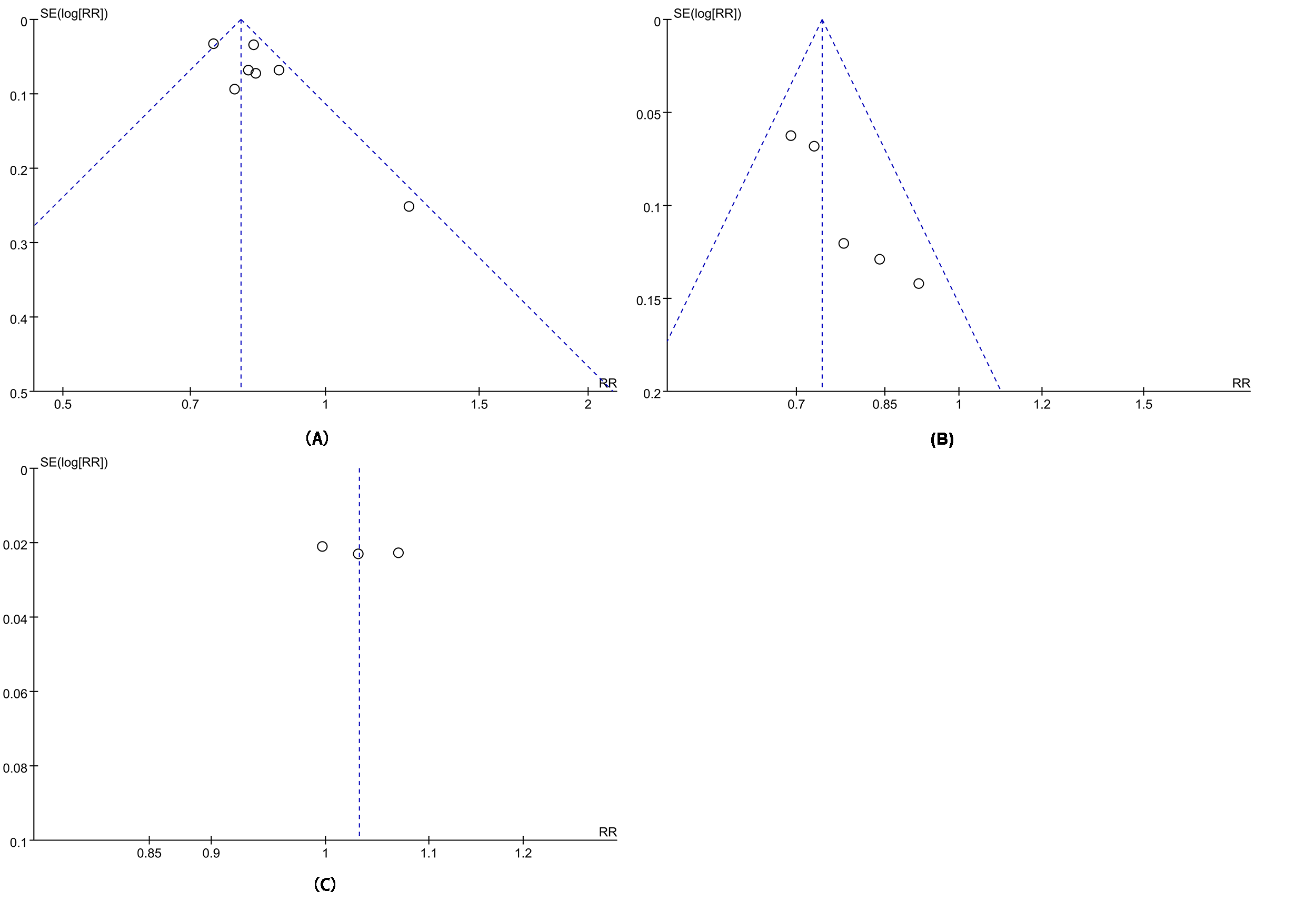


**Fig S2.** Funnel plots of outcome indicator. **(A)** incidence of CRC; **(B)**mortality of CRC; **(C)** mortality for all causes.


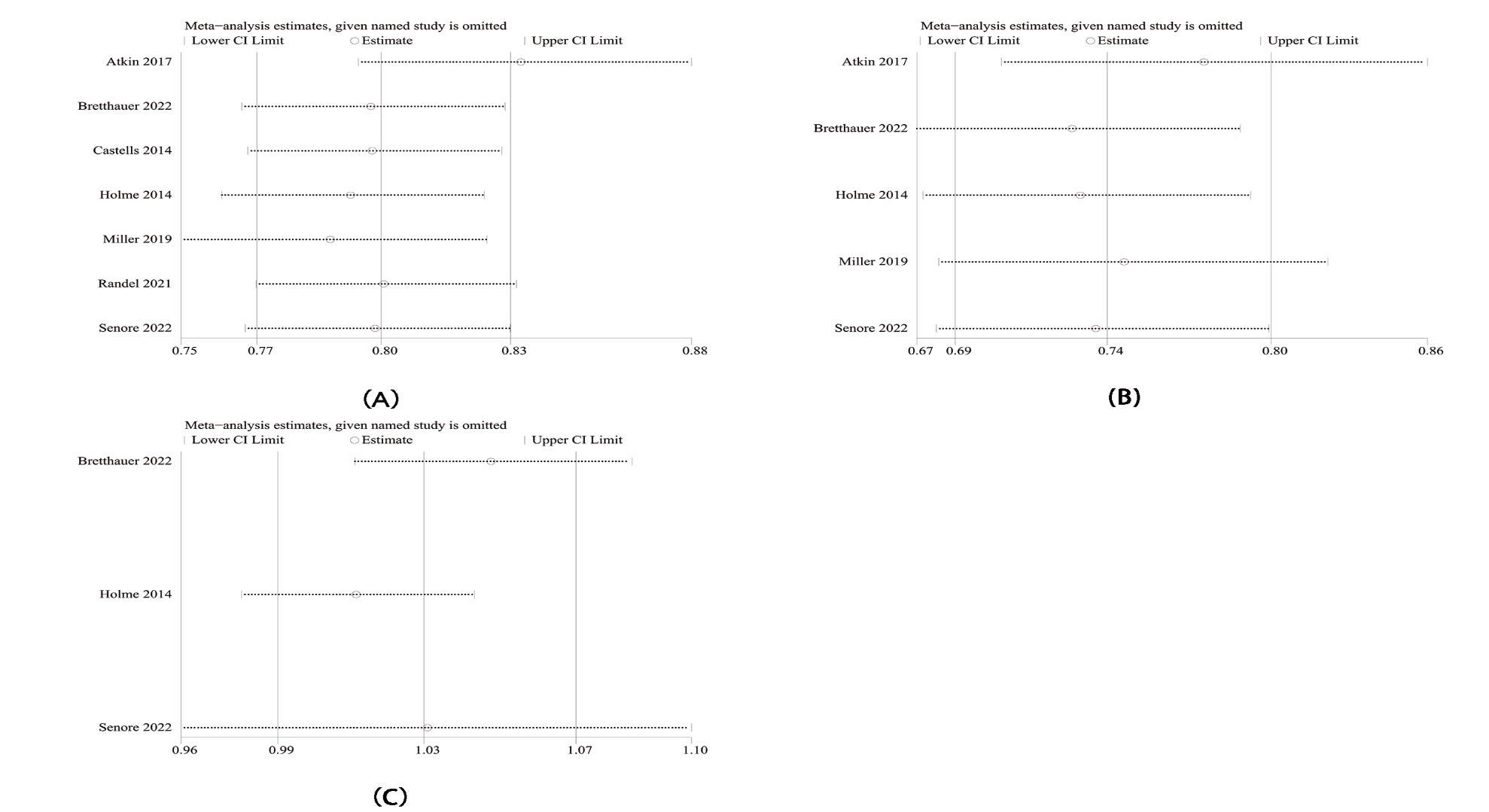


**Fig S3.** Sensitivity analysis of **(A)** incidence of CRC, **(B)**mortality of CRC, **(C)** mortality for all causes.
